# Comprehensive Simulation-Based Evaluation of Gamma Radiation Shielding Performance of Bismuth Oxide- and Tungsten Oxide-Reinforced Polymer Composites for Nuclear Medicine Occupational Safety

**DOI:** 10.3390/polym17111491

**Published:** 2025-05-27

**Authors:** Suphalak Khamruang Marshall, Poochit Kwandee, Nueafa Songphum, Jarasrawee Chuaymuang

**Affiliations:** Department of Radiology, Faculty of Medicine, Prince of Songkla University, Songkhla 90110, Thailand

**Keywords:** bismuth oxide, gamma shielding, Monte Carlo simulation, Phy-X/PSD, polymer composites, radiation attenuation, radiation protection, shielding materials, tungsten oxide, XCOM simulation

## Abstract

This study employs simulation tools to design and evaluate lightweight, lead-free polymer composites incorporating polytetrafluoroethylene (PTFE), polyethylene (PE), and polyetherimide (PEI) for gamma radiation shielding in nuclear medicine. Targeting clinically relevant photon energies from Tc-99m (140 keV), I-131 (364 keV), and Cs-137 (662 keV), composites’ structural and shielding performance with Bi_2_O_3_ and WO_3_ was assessed using XCOM and Phy-X/PSD. PEI emerged as the most suitable polymer for load-bearing and thermally exposed applications, offering superior mechanical stability and dimensional integrity. Bi_2_O_3_-WO_3_ fillers for Tc-99m achieved a ~7000-fold increase in MAC, I-131 ~2063-fold, and Cs-137 ~1370-fold compared to PbO_2_. The PEI-75Bi_2_O_3_-25WO_3_ achieved a ~21-fold reduction in half-value layer (HVL) compared to lead for Tc-99m. For higher-energy isotopes of I-131 and Cs-137, HVL reductions of ~0.44-fold and ~0.08-fold, respectively, were achieved. The results demonstrate that high-Z dual filler polymer composites have an equal or enhanced attenuation properties to lead-based shielding, whilst also enhancing the polymer composites’ mechanical and thermal characteristics. As the use of ionizing radiation increases, so does the potential risks; high-Z dual filler polymer composites provide a sustainable, lightweight, non-toxic alternative to conventional lead shielding.

## 1. Introduction

The medical and scientific communities have long recognized radiation and radioactive materials’ diagnostic and therapeutic potential. Roentgen made the first X-ray discovery in 1895 [1], and shortly after the first application of radiation for cancer was in 1896 [2]. Thereafter, the discipline quickly developed, and by the 1930s, it became formalized as a specialty. Since then, our knowledge of radiation interactions with biological tissues has developed significantly, and consequently its therapeutic applications expanded as a result of developments in radiation research [3,4,5,6,7,8]. Moreover, medical imaging plays an essential role in diagnostics and treatment, enabling the early identification, therapy planning, medication distribution, and response monitoring of medical issues [9]. Consequently, the utilization of ionizing radiation in healthcare has increased. However, there is growing concern regarding the cumulative exposure to patients and healthcare personnel due to the rising usage of ionizing radiation [10,11]. Of note, 1.33% of CT patients are exposed to a cumulative effective dose (CED) of more than 100 mSv [12], and the present rate of CT use may cause more than 103,000 cases of radiation-induced malignancies each year, accounting for up to 5% of all new cancer diagnoses [13].

Medical radiologists are routinely exposed to low-dose ionizing radiation (LDIR) due to their occupational environment [14]. They can take confidence in the fact that protective measures are not only in place but also standardized. However, it is crucial to be informed that chronic, cumulative exposure may lead to systemic biological effects, including metabolic disruptions. One such adverse outcome increasingly observed in this population is dyslipidemia, particularly characterized by elevated triglycerides. Wen et al. reported that medical radiologists have a high rate of dyslipidemia and that an increasing cumulative radiation effective dose increases the risk of dyslipidemia and increased triglycerides [15]. Notably, dyslipidemia is also known as a fundamental risk factor for cardiovascular diseases [16]. Moreover, Picano et al. reported an updated analysis of radiation-induced health risks, particularly emphasizing the elevated incidence of cardiovascular disease and cancer correlated with an increasing use of diagnostic imaging procedures. The authors claim that the average yearly per capita radiation dosage from medical imaging in the United States has risen significantly to 2.29 mSv in 2016 from 0.50 mSv in 1996. This represents a 4.58-fold increase, signifying a greater societal health burden than previously recognized [17].

Although lead has significant radiation shielding qualities, with a high atomic number (Z = 82) and density (11.34 g/cm^3^), making it effective in attenuating X-rays and gamma rays, it also has drawbacks restricting its widespread use. A study by Hout, Jason D., and JuHyeong Ryu determined that healthcare staff wearing heavy lead aprons were associated with a significantly higher probability of injury. Other restrictions include toxicity, structural constraints, environmental issues, and recycling and disposal issues [18]. The environmental effect of lead is considerable, and correct disposal must be carried out to avoid soil and water contamination [19]. Improper disposal endangers human health and ecosystems. Alternative shielding materials such as bismuth oxide and tungsten oxide are less toxic and easier to dispose of, lessening environmental risks. The World Health Organization (WHO) has listed lead in the top ten chemicals or groups of chemicals of health concern. Furthermore, exposure to lead is associated with cardiovascular effects, neurotoxicity, and renal dysfunction [20]. Additionally, Larsen and Sánchez-Triana projected the lead exposure worldwide health load to be similar to that of PM2.5 air pollution [21].

As a result, more scientific studies have examined these problems associated with lead, pursuing more sustainable and practicable radiation shielding substitutes. For example, polymers for radiation shielding have drawn significant interest, particularly in fields such as medicine, nuclear energy, and aerospace, as a result of increasing demand for non-toxic and lightweight alternative materials [22,23]. Polymer composites are especially appealing due to their low mass, ease of fabrication, and capacity for chemical modification, offering a versatile alternative to traditional shielding substances. However, in their pure form polymers are generally poor attenuators of ionizing radiation such as gamma rays and X-rays due to their low atomic number (Z) and density. On the other hand, when modified with high-Z elements or nanoparticles—such as bismuth (Z = 83), tungsten (Z = 74), or lead (Z = 82)—they can become highly effective at radiation shielding [24]. The principle behind this enhancement lies in the photoelectric effect, a process where high-Z atoms increase the probability of photon interaction, leading to greater energy absorption and attenuation.

The originality of this study is the first-time evaluation of dual high-Z fillers in PTFE, PE, and PEI polymer matrices shielding performance and structural properties. Typically, former studies have concentrated on single metal oxides reinforced with a single polymer. This study builds upon our previous studies assessing the attenuation properties of tungsten oxide-reinforced polyisoprene [25] and bismuth oxide nanoparticle-enhanced poly(methyl methacrylate) composite [26]. The polymer matrices were selected based on key criteria for radiation shielding resistance to gamma-induced chemical and structural degradation, thermal and mechanical stability, and compatibility with high-Z fillers like Bi_2_O_3_ and WO_3_ for uniform dispersion and effective attenuation, and ease of processing into practical shielding forms. Herein, we specifically evaluate the attenuation characteristics of PTFE-WO_3_, PE-WO_3_, PEI-WO_3_, PTFE-Bi_2_O_3_, PE-Bi_2_O_3_, PEI-Bi_2_O_3_, PTFE-Bi_2_O_3_-WO_3_, PE-Bi_2_O_3_-WO_3_, and PEI-Bi_2_O_3_-WO_3_ against three clinically relevant radionuclides: technetium-99m (140 keV), iodine-131 (364 keV), and cesium-137 (662 keV). The rationale behind the selection of PTFE, PE, and PEI as the polymer matrices for our radiation shielding composites, is on a combination of radiation resistance, thermal and mechanical stability, compatibility with high-Z fillers (bismuth oxide and tungsten oxide), and processability. Each polymer brings unique advantages: PTFE provides high density and chemical resistance. PE is rich in hydrogen, contributing to gamma and neutron scattering and is lightweight and easy to fabricate. PEI provides high mechanical strength and radiation resistance, making it suitable for high-performance applications.

## 2. Materials and Methods

### 2.1. Materials

Each selected polymer contributes uniquely to the performance of the composite system. PTFE was chosen for its high density (~2.2 g/cm^3^), excellent chemical resistance, and outstanding thermal stability. These properties make PTFE an ideal candidate for maintaining structural and chemical integrity under irradiation, particularly in demanding environments. Furthermore, its relatively high density among polymers contributes favorably to gamma attenuation effectiveness.

PE, on the other hand, offers high hydrogen content, which is advantageous for gamma and neutron shielding through scattering interactions. PE is also lightweight, cost-effective, and easily processable, making it a practical choice for the production of shielding components where fabrication simplicity and material economy are important considerations. Its well-established use in existing shielding applications further supports its selection.

PEI is a high-performance thermoplastic characterized by superior mechanical strength, excellent thermal stability (glass transition temperature ~215 °C), and intrinsic flame retardance. Importantly, PEI demonstrates good resistance to radiation-induced degradation, making it suitable for use in high-demand environments such as aerospace, medical, and nuclear facilities. Its ability to retain structural integrity under both thermal and radiative stress underscores its value in multifunctional shielding systems practical deployment.

### 2.2. ANSYS Simulation of Material Behavior in Bi_2_O_3_- and WO_3_-Reinforced Polymer Composites

#### 2.2.1. Heat Transfer

To evaluate the thermal performance of polymer composites for radiation shielding applications, a heat transfer simulation was conducted on three types of polymer matrices: polytetrafluoroethylene (PTFE), polyethylene (PE), and polyetherimide (PEI). The simulation was performed using ANSYS Workbench 2025 R1 (version 25.1) (Ansys, Inc., Canonsburg, PA, USA), a commercial finite element analysis (FEA) platform. The heat transfer module was used to simulate transient thermal conduction within a standard rectangular block (dimensions: 80 mm × 40 mm × 10 mm). The initial temperature of the entire material domain was set to 22 °C (room temperature). One face of the block was exposed to a constant temperature equal to the respective melting point of each matrix material, simulating heat exposure during operational conditions. All other surfaces were treated as thermally insulated boundaries. Each simulation was run over a duration of 30 min to observe thermal diffusion through the material. The primary output was the temperature distribution along the *x*-axis (depth), with 3D contour plots generated to visualize heat penetration.

#### 2.2.2. Bending Behavior

To assess the deformation behavior of Bi_2_O_3_- and WO_3_-reinforced polymer composites including PTFE, PE, and PEI matrices, a structural bending study was carried out. These composites were developed to investigate their mechanical reaction under different force applications, therefore imitating possible mechanical stresses seen in actual radiation shielding applications. Using ANSYS Workbench 2025 R1 (version 25.1) (Ansys, Inc., Canonsburg, PA, USA), the simulation research was run on an integrated platform for advanced FEA.

ANSYS DesignModeler [27] was used to model the composite sheet in three dimensions (3D). To reflect a reasonable size for composite sheet applications, the geometrical parameters of the model were set at 80 mm in length, 40 mm in width, and 10 mm in thickness. A structured mesh was created with sufficient refinement to ensure convergence and correctness in the findings, hence attaining high fidelity in capturing stress–strain distribution and deformation profiles. Furthermore mesh sensitivity analysis helped to improve element size and meshing density, hence balancing computational economy with accuracy in important areas of stress concentration.

Material properties were meticulously assigned based on a combination of experimental data and validated literature values [28,29,30,31,32,33]. These included the elastic modulus, Poisson’s ratio, and density specific to each polymer matrix and reinforced composite formulation. The properties of Bi_2_O_3_ and WO_3_ fillers were integrated according to their respective weight fractions within the composite matrices, accurately reflecting the heterogeneous nature of the materials. Boundary conditions were defined to replicate experimental bending scenarios. The bottom edge of the composite sheet was fully fixed, restraining all translational and rotational degrees of freedom to simulate a rigid support. A concentrated point load was applied vertically along the *y*-axis near the center of the upper surface of the sheet, representing the primary bending load direction. The selection of the loading point was based on standard three-point bending configurations to induce maximal bending stress at mid-span.

To systematically examine the mechanical response under different stress levels, force magnitudes were incrementally varied across four defined conditions: 10 N, 25 N, 50 N, and 100 N. The lower loads (10 N and 25 N) reflect light mechanical stresses typically encountered during handling, assembly, or incidental contact. The higher loads (50 N and 100 N) represent moderate to significant operational stresses, such as sustained pressure from mounted equipment or structural loads in wall-mounted, suspended, or mobile shielding systems. This load range was chosen to assess both elastic and nonlinear deformation responses, enabling a comparative evaluation of the mechanical stability and structural reliability of the polymer composites under conditions relevant to real-world applications. The analysis employed a nonlinear static structural solution approach, capable of capturing large deformation effects and material nonlinearities that may arise under elevated loading. Quasi-static loading conditions were assumed, given that the deformation process occurs at sufficiently slow rates to neglect dynamic effects. Contact formulations, if present at interfaces, were treated as bonded to simulate perfect adhesion between phases. For each loading scenario, the maximum displacement was recorded at the point of force application. Additionally, the stress distribution and strain patterns were analyzed to understand load transfer mechanisms within the composite structure. Results were extracted in graphical and tabular formats for comprehensive comparison across materials and loading conditions, enabling a detailed evaluation of the structural integrity and deformation tolerance of each composite formulation.

This computational approach provided predictive insights into the mechanical performance of Bi_2_O_3_- and WO_3_-reinforced polymer composites under bending loads. The findings contribute to the broader objective of developing multifunctional shielding materials that combine effective radiation attenuation with mechanical robustness, essential for reliable performance in occupational radiation protection systems.

### 2.3. Simulation of Photon Interaction Cross-Sections in High-Z Reinforced Polymers Using XCOM for Occupational Radiation Protection

In this study, the XCOM photon cross-section database, established by the National Institute of Standards and Technology (NIST), was utilized to compute the fundamental radiation interaction parameters of the materials under investigation. Specifically, MAC and partial interaction cross-sections were calculated for Compton incoherent scattering, pair production in the nuclear field, and photoelectric absorption. Additionally, the total attenuation coefficients were determined without the contribution of coherent scattering. These values provide comprehensive insight into the photon interaction behavior of composite materials reinforced with high-Z fillers, such as Bi_2_O_3_ and WO_3_.

The simulation outcomes were systematically compared against reference data from the Evaluated Photon Interaction Cross Sections (EPICS2017) database [34,35] to validate the accuracy of the computed results. XCOM computes total and partial photon interaction cross-sections based on fundamental physical models and experimental data compilations, applying interpolation across a broad energy spectrum. Calculations were performed over an energy range spanning from 1 keV to 100 MeV, using a logarithmically spaced energy grid to ensure fine resolution across low to high photon energies. This approach enables detailed characterization of material behavior across the full range of photon energies relevant to nuclear medicine applications.

For ease of material specification and efficient workflow, the Windows-based graphical user interface (GUI) version of XCOM was employed [36]. This interface facilitated the accurate input of compound formulations by allowing the selection of individual elements and their respective weight fractions, which is particularly advantageous for evaluating complex multi-phase composites such as Bi_2_O_3_-WO_3_ polymer matrices. The calculated MAC (cm^2^/g) were extracted and subsequently analyzed to assess the effectiveness of each composite in attenuating photon radiation at specific energies corresponding to clinically relevant radionuclides, including Tc-99m, I-131, and Cs-137.

Overall, the use of XCOM provided a robust and validated computational framework for predicting the photon attenuation behavior of advanced shielding materials. These calculations underpin the comparative analysis of different composite formulations, contributing to the rational design and optimization of high-performance radiation shielding materials for occupational safety in nuclear medicine environments.

### 2.4. Comprehensive Phy-X/PSD Computational Analysis of Gamma Radiation Attenuation Parameters in Bi_2_O_3_- and WO_3_-Reinforced Polymer Composites for Enhanced Occupational Radiation Shielding in Nuclear Medicine

The radiation shielding properties of a range of polymer composites and metal oxide formulations were comprehensively evaluated using the Phy-X/PSD computational simulation [37]. This advanced tool enables the calculation of photon interaction parameters across a wide energy spectrum, providing an in-depth assessment of material performance for radiation protection applications. The materials analyzed in this study included individual compounds (PbO_2_, PTFE, PE, PEI, Bi_2_O_3_, and WO_3_), binary composites (Bi_2_O_3_-WO_3_), and various reinforced polymer composites, specifically PTFE-WO_3_, PE-WO_3_, PEI-WO_3_, PTFE-Bi_2_O_3_, PE-Bi_2_O_3_, PEI-Bi_2_O_3_, PTFE-Bi_2_O_3_-WO_3_, PE-Bi_2_O_3_-WO_3_, and PEI-Bi_2_O_3_-WO_3_.

The user-friendly interface of Phy-X/PSD facilitated the accurate definition of composite formulations by allowing precise input of elemental compositions and weight fractions, which is particularly beneficial when analyzing complex multiphase systems such as Bi_2_O_3_-WO_3_ reinforced polymers. The key shielding parameters were systematically calculated to provide a comprehensive profile of the materials’ radiation attenuation capabilities. These parameters included the linear attenuation coefficient (LAC), which accounts for the influence of material density on photon attenuation; the half-value layer (HVL) and tenth-value layer (TVL), representing the material thicknesses required to reduce photon intensity by 50% and 90%, respectively; and the mean free path (MFP), indicating the average distance a photon travels before interacting with the material. These values were carefully extracted and analyzed to evaluate the effectiveness of each composite in attenuating gamma photons at energies relevant to nuclear medicine applications, notably those emitted by Tc-99m, I-131, and Cs-137.

Therefore, simulations were performed over an energy range of 1 keV to 10 MeV to encompass the full spectrum of photon energies relevant to both diagnostic and therapeutic nuclear medicine procedures. All input data reflected the precise elemental compositions and weight fractions of the selected composites. The resulting data were exported in tabular format for comparative analysis, enabling the identification of optimal material formulations for enhanced radiation shielding effectiveness. Through this comprehensive computational approach, the study systematically evaluated the photon attenuation behaviors of a diverse set of composites. These findings contribute valuable insights for the design and development of advanced, and efficient shielding materials aimed at improving occupational radiation protection in nuclear medicine settings.

### 2.5. Statistical Analysis

Statistical analyses were performed using the Student’s *t*-test and either one-way or two-way analysis of variance (ANOVA), followed by the Student–Newman–Keuls post hoc test to evaluate differences between experimental groups. Statistical significance was defined as *p* < 0.05 (denoted by *), and high significance as *p* < 0.01 (denoted by **), while values of *p* > 0.05 were considered not significant (denoted by ns). All statistical procedures were conducted using GraphPad Prism software, version 10.0 (GraphPad Software Inc., Boston, MA, USA).

## 3. Results

### 3.1. Finite Element Analysis of Heat Transfer and Bending-Induced Structural Deformation in Bismuth Oxide and Tungsten Oxide-Reinforced Polymer Composites Using ANSYS

#### 3.1.1. Numerical Simulation of Heat Transfer and Material Performance in ANSYS

**Figure 1:**
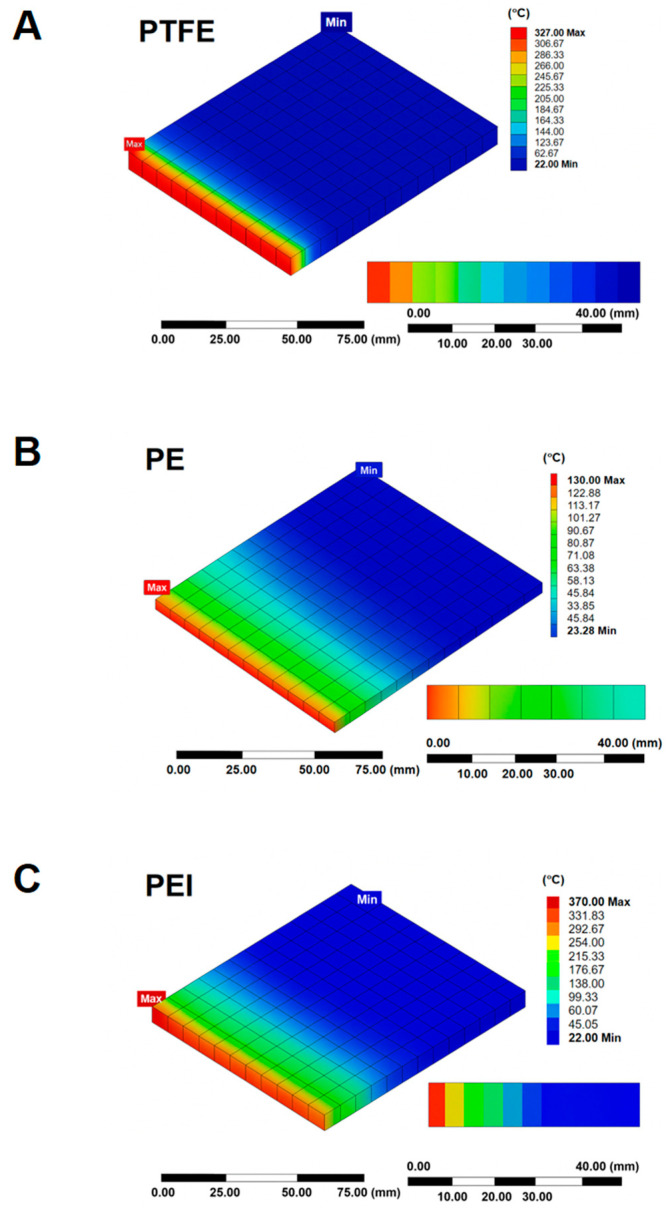
Simulated heat transfer profiles of three polymeric materials subjected to their respective melting point temperatures for 30 min: (**A**) polytetrafluoroethylene (PTFE), (**B**) polyethylene (PE), and (**C**) polyetherimide (PEI). The color gradient represents temperature distribution (°C), highlighting the extent of thermal penetration.

Figure 1 presents the simulated heat transfer profiles of three polymeric materials—PTFE, PE, and PEI—subjected to their respective melting point temperatures over a 30-min duration. The temperature distributions provide valuable insights not only into the thermal conductivity and heat penetration characteristics of each material but also their potential suitability as matrices in gamma radiation shielding composites for occupational use in nuclear medicine.

When considering the integration of high-Z fillers such as Bi_2_O_3_ or WO_3_ into polymer matrices to produce flexible and lightweight radiation shields, the thermal stability of the base polymer becomes an important factor. Among the three materials, PE (Figure 1B), which has a relatively low melting point of approximately 130 °C, exhibits extensive heat penetration. Temperatures above 120 °C reached depths up to 6 mm, indicating rapid heat transfer and low thermal resistance. While this makes PE favorable for low-temperature processing and molding of composites, it may be less suitable for environments with prolonged exposure to heat, such as those encountered during sterilization or continuous use near radiation-emitting sources.

In contrast, PTFE (Figure 1A) and PEI (Figure 1C), with melting points near 327 °C and 370 °C respectively, exhibit limited heat penetration (approximately 2 mm), suggesting higher thermal resistance and structural integrity under thermal stress. These characteristics are desirable for shielding materials that must maintain dimensional stability and protective properties under elevated temperatures or when integrated into protective garments worn for extended durations in nuclear medicine facilities.

Therefore, when selecting a polymer matrix for gamma radiation shielding applications in clinical environments, a balance must be achieved between thermal stability, processing ease, and compatibility with high-density fillers. PE may be preferable in disposable or short-use shields due to ease of fabrication, whereas PTFE and PEI are more appropriate for reusable, high-durability protective equipment. The enhanced thermal resistance of PTFE and PEI may also help prevent filler degradation and maintain uniform distribution, ensuring consistent shielding effectiveness over time.

#### 3.1.2. Bending-Induced Structural Deformation

**Figure 2:**
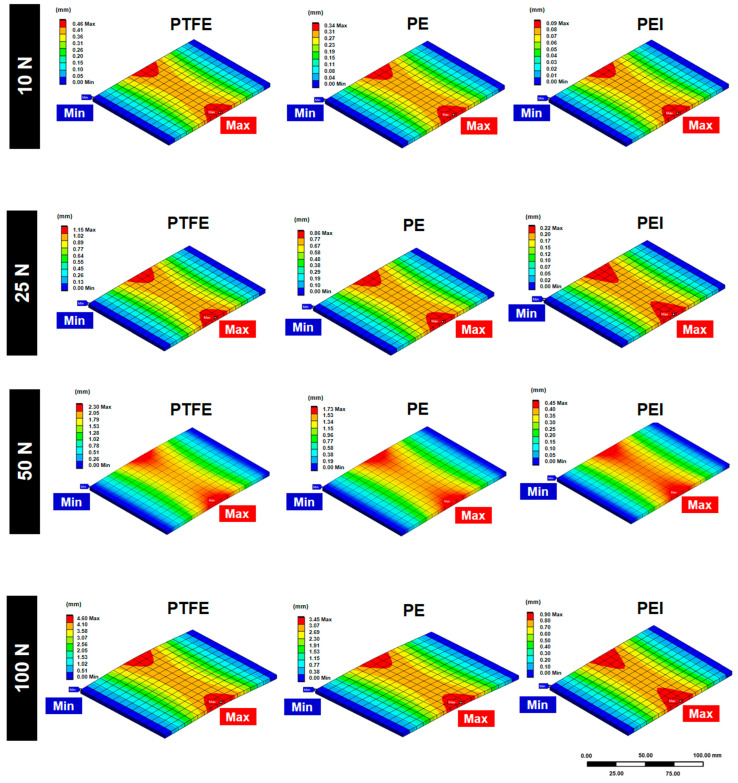
ANSYS simulation of material behavior under bending deformation for three polymeric materials: polytetrafluoroethylene (PTFE), polyethylene (PE), and polyetherimide (PEI). The finite element analysis (FEA) was performed under compressive loads of 10 N, 25 N, 50 N, and 100 N. The color contour plots represent the resulting vertical displacement (in mm).

This study employed finite element analysis via ANSYS Workbench 2025 R1 (version 25.1) (Ansys, Inc., Canonsburg, PA, USA) to evaluate the bending deformation behavior of three polymeric materials: PTFE, PE, and PEI under four levels of compressive force: 10 N, 25 N, 50 N, and 100 N (Figure 2). The primary objective was to assess the structural stability and mechanical deformation of these polymers in the context of potential applications in radiation shielding systems, where dimensional integrity under mechanical stress is critical for long-term protection efficacy and safety.

At the lowest load condition (10 N), PTFE demonstrated the highest deformation with a maximum vertical displacement of 0.46 mm, followed by PE at 0.34 mm, and PEI at only 0.09 mm. This early result highlights the superior stiffness and resistance to bending of PEI, which is essential in radiation shielding applications that involve modular panels or protective enclosures subject to compressive or bending loads. PE exhibited moderate flexibility, whereas PTFE’s relatively large deformation at minimal force raises concerns about long-term dimensional stability in protective structures.

When the applied force was increased to 25 N, the trend remained consistent. PTFE deformed by up to 1.15 mm, PE by 0.86 mm, and PEI by only 0.22 mm. As the structural integrity of shielding materials is vital for maintaining consistent attenuation of ionizing radiation, excessive bending or warping could result in gaps or misalignments between protective panels, compromising their shielding performance. In this regard, PEI maintained its low-displacement behavior and structural integrity across the increased load range.

Under a 50 N force, PTFE and PE displayed further increases in deformation (2.30 mm and 1.73 mm, respectively), whereas PEI only reached 0.45 mm. This indicates that while PE may be acceptable for flexible shielding components or temporary applications, it may not be suitable for structural elements requiring rigid alignment and durability. PTFE, although offering some benefits in terms of chemical inertness and thermal stability, presents challenges due to its mechanical softness, which may lead to progressive deformation under sustained load—particularly in vertical or weight-bearing installations.

At the maximum load of 100 N, the disparities between materials became more pronounced. PTFE reached a maximum displacement of 4.60 mm, while PE deformed up to 3.45 mm. PEI, on the other hand, maintained deformation below 1 mm (0.90 mm), demonstrating remarkable mechanical robustness. In shielding scenarios involving wall-mounted panels, ceiling suspensions, or mobile barrier systems, deformation on this scale can directly impact the continuity and alignment of shielding coverage. Therefore, PEI offers a critical advantage in maintaining the designed shielding geometry, ensuring consistent radiation attenuation.

Overall, the results confirm that PEI possesses superior mechanical stiffness and dimensional stability under compressive loading compared to PTFE and PE. This mechanical advantage is crucial in radiation shielding applications, where material deformation could result in compromised coverage, misalignment with adjoining components, or long-term mechanical fatigue under repeated use. While PE may still be appropriate for lightweight or secondary shielding structures, and PTFE may be useful in flexible joints or chemically resistant layers, PEI is the most suitable candidate for load-bearing or structurally exposed shielding components. Furthermore, the linear and predictable deformation behavior of PEI and PE under increasing loads supports their integration into systems where load forecasting and modeling are essential. In contrast, the nonlinear behavior of PTFE under higher loads may necessitate additional safety factors in design or the use of reinforcement to maintain shielding efficacy. These insights contribute to improved material selection for radiation protection equipment, particularly in clinical, industrial, and research environments where mechanical durability under load is as critical as attenuation performance.

### 3.2. XCOM-Based Computational Analysis of Mass Attenuation Coefficients in Bismuth Oxide and Tungsten Oxide-Reinforced Polymer Composites for Gamma Radiation Shielding

#### 3.2.1. Incoherent Scattering

**Figure 3:**
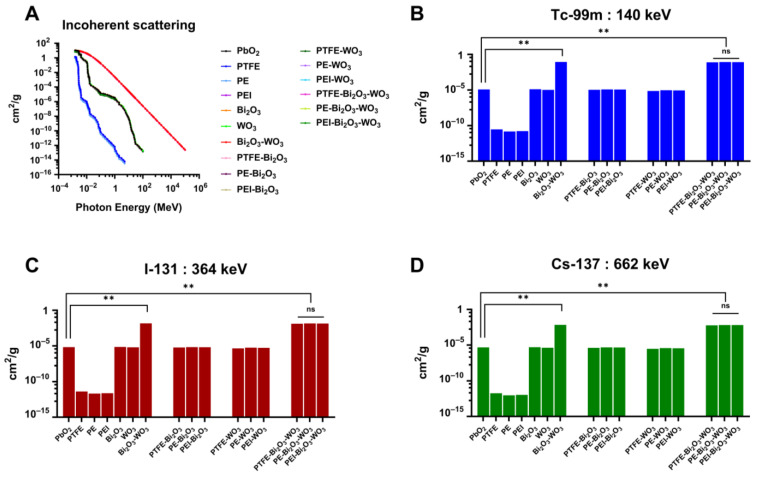
XCOM-based computational analysis of mass attenuation coefficients (cm^2^/g) for incoherent scattering in bismuth oxide and tungsten oxide-reinforced polymer composites for gamma radiation shielding. (**A**) Comparison of incoherent scattering cross-sections as a function of photon energy (MeV) for various materials including PbO_2_, PTFE, PE, PEI, Bi_2_O_3_, WO_3_, Bi_2_O_3_-WO_3_ composite, and reinforced polymer composites (PTFE-WO_3_, PE-WO_3_, PEI-WO_3_, PTFE-Bi_2_O_3_, PE-Bi_2_O_3_, PEI-Bi_2_O_3_, PTFE-Bi_2_O_3_-WO_3_, PE-Bi_2_O_3_-WO_3_, PEI-Bi_2_O_3_-WO_3_). (**B**–**D**) Bar graphs showing mass attenuation coefficients for incoherent scattering at specific photon energies: (**B**) Tc-99m (140 keV), (**C**) I-131 (364 keV), and (**D**) Cs-137 (662 keV). Statistical significance is denoted as ** *p* < 0.01; results are considered not significant when *p* > 0.05 (ns).

The MAC for incoherent (Compton) scattering was computed using XCOM simulations for a range of polymer composites reinforced with Bi_2_O_3_ and WO_3_, alongside pure polymers and PbO_2_ as a reference material (Figure 3).

Figure 3B presents the MAC (cm^2^/g) of various materials and their composites at a photon energy of 140 keV from Tc-99m, focusing on incoherent scattering. Compared to PbO_2_, a conventional benchmark material for gamma radiation shielding, the PEI-Bi_2_O_3_-WO_3_ composite demonstrates a remarkable improvement in MAC. While PbO_2_ has a MAC of 1.19 × 10^−5^ cm^2^/g, the PEI-Bi_2_O_3_-WO_3_ composite reaches 8.32 × 10^−2^ cm^2^/g at 140 keV. This represents a ~7000-fold increase in attenuation efficiency. Such a substantial enhancement highlights the advantage of incorporating high-Z materials like bismuth oxide and tungsten oxide into polymer matrices.

At a higher photon energy of 364 keV from I-131 (Figure 3C), the PEI-Bi_2_O_3_-WO_3_ composite continues to outperform traditional shielding materials. While PbO_2_ shows a MAC of 6.69 × 10^−6^ cm^2^/g, the PEI-Bi_2_O_3_-WO_3_ composite reaches 1.38 × 10^−2^ cm^2^/g, corresponding to an approximate 2063-fold increase in attenuation efficiency. This performance boost highlights the superior gamma shielding capabilities of the composite, particularly in the context of incoherent scattering at higher energies. Despite the general trend of reduced attenuation with increasing energy, the composite material retains a strong shielding effect, indicating robust energy-independent performance.

At a photon energy of 662 keV from Cs-137 (Figure 3D), the PEI-Bi_2_O_3_-WO_3_ composite again significantly outperforms traditional PbO_2_. The MAC for PbO_2_ is 4.65 × 10^−6^ cm^2^/g, whereas the PEI-Bi_2_O_3_-WO_3_ composite achieves 6.37 × 10^−3^ cm^2^/g, resulting in approximately a 1370-fold improvement. Although the overall MAC decreases at higher energies—a typical trend due to reduced interaction probability at higher photon energies—the composite material retains superior shielding efficiency. This consistent outperformance across various gamma energies confirms the energy-independent shielding potential of the PEI-Bi_2_O_3_-WO_3_ system.

As a result, the results noticeably indicate that the PEI-Bi_2_O_3_-WO_3_ composite exhibits significantly superior gamma radiation shielding capabilities in comparison to conventional PbO_2_ across all examined photon energies. The composite consistently demonstrated 1370- to nearly 7000-fold higher MAC, depending on energy, highlighting its effectiveness not only at low-energy emissions like Tc-99m (140 keV) but also at higher-energy sources such as I-131 (364 keV) and Cs-137 (662 keV). This performance, combined with the material’s potential benefits in terms of flexibility, reduced toxicity, and lower weight, establishes it as a robust and viable lead-free alternative for modern radiation shielding applications.

#### 3.2.2. Photoelectric Absorption

**Figure 4:**
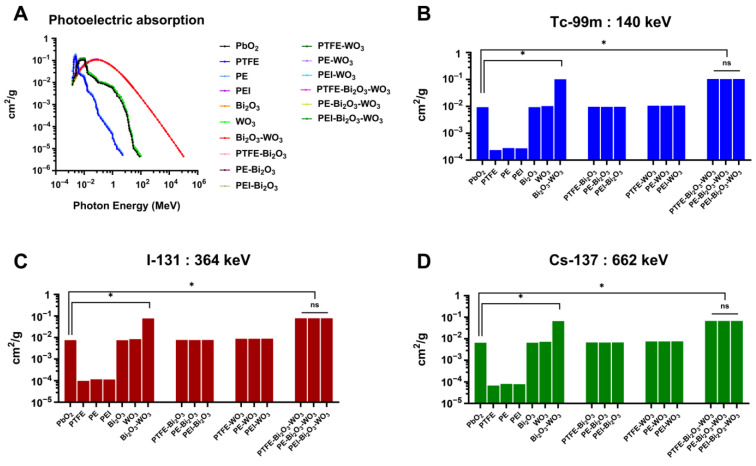
XCOM-based computational analysis of mass attenuation coefficients (cm^2^/g) for photoelectric absorption in bismuth oxide and tungsten oxide-reinforced polymer composites for gamma radiation shielding. (**A**) Comparison of photoelectric absorption cross-sections as a function of photon energy (MeV) for various materials including PbO_2_, PTFE, PE, PEI, Bi_2_O_3_, WO_3_, Bi_2_O_3_-WO_3_ composite, and reinforced polymer composites (PTFE-WO_3_, PE-WO_3_, PEI-WO_3_, PTFE-Bi_2_O_3_, PE-Bi_2_O_3_, PEI-Bi_2_O_3_, PTFE-Bi_2_O_3_-WO_3_, PE-Bi_2_O_3_-WO_3_, PEI-Bi_2_O_3_-WO_3_). (**B**–**D**) Bar graphs showing mass attenuation coefficients for photoelectric absorption at specific photon energies: (**B**) Tc-99m (140 keV), (**C**) I-131 (364 keV), and (**D**) Cs-137 (662 keV). Statistical significance is denoted as * *p* < 0.05; results are considered not significant when *p* > 0.05 (ns).

Figure 4A illustrates the photoelectric absorption cross-sections as a function of photon energy. All materials display a decreasing trend in absorption with increasing photon energy, consistent with the photoelectric effect, which predominates at lower photon energies. Among the materials examined, PbO_2_ exhibits the highest absorption cross-section throughout the energy range, a performance attributed to its high atomic number and density, which facilitate greater photon-matter interactions. Comparatively, unfilled polymers such as PTFE, PE, and PEI show significantly lower absorption capacities. However, the incorporation of Bi_2_O_3_ markedly enhances the photoelectric absorption of these polymers, owing to the high atomic number of bismuth. The effect is further amplified when Bi_2_O_3_ is combined with WO_3_ to form hybrid composites. Notably, these Bi_2_O_3_-WO_3_ hybrid systems maintain superior absorption efficiencies in the lower photon energy region (below 1 MeV), suggesting a synergistic interaction between the two fillers that augments the overall attenuation capacity.

Figure 4B illustrates the photoelectric absorption cross-sections for various shielding materials at 140 keV, where the photoelectric effect is the dominant interaction mechanism. Among all the tested samples, the PEI-Bi_2_O_3_-WO_3_ composite exhibits the highest attenuation value of 0.10 cm^2^/g, while the conventional benchmark material PbO_2_ records a significantly lower value of 9.34 × 10^−3^ cm^2^/g. Indicating the composite demonstrates an approximately 11-fold improvement in photoelectric absorption over PbO_2_ at this diagnostic energy level. The enhanced performance can be attributed to the synergistic effect of combining two high-Z oxides—Bi_2_O_3_ and WO_3_—within a polymer matrix (PEI), maximizing photon interaction probability while maintaining a lightweight and flexible form.

The photoelectric absorption cross-sections of various materials at 364 keV (Figure 4C) illustrates the intermediate photon energy representative of I-131 emissions. At this energy, the dominant interaction mechanism shifts from photoelectric absorption toward Compton scattering, leading to generally lower attenuation values compared to lower energies. Despite this, the PEI-Bi_2_O_3_-WO_3_ composite demonstrates a strong shielding performance with a MAC of 7.84 × 10^−2^ cm^2^/g, significantly surpassing PbO_2_, which registers at 7.50 × 10^−3^ cm^2^/g, which translates to a 10.45-fold increase in radiation attenuation efficiency. These findings support the superior and consistent performance of PEI-Bi_2_O_3_-WO_3_ composites, making them ideal for applications requiring shielding against medium-energy gamma radiation, such as I-131 in nuclear medicine and radiotherapy environments.

At a photon energy of 662 keV, representative of emissions from Cs-137 (Figure 4D), PbO_2_ demonstrates an attenuation coefficient of 6.51 × 10^−3^ cm^2^/g. At the same time, while the PEI-Bi_2_O_3_-WO_3_ composite achieves a substantially higher value of 6.70 × 10^−2^ cm^2^/g, indicating a ~10.30-fold increase in gamma attenuation efficiency. This performance highlights the composite’s superior shielding capacity even at elevated photon energies, where photoelectric effects have diminished influence. Despite the general decline in mass attenuation values at higher energies, the PEI-Bi_2_O_3_-WO_3_ composite retains its effectiveness, primarily due to the presence of high-Z elements that contribute meaningfully to Compton scattering.

These findings highlight the potential impact of PEI-Bi_2_O_3_-WO_3_ composites as highly effective, lead-free gamma radiation shielding materials across a broad range of photon energies. From low-energy emissions typical of diagnostic imaging (Tc-99m, 140 keV) to intermediate and high-energy sources used in therapy (I-131, 364 keV; Cs-137, 662 keV), the composite consistently outperforms traditional PbO_2_. With attenuation improvements ranging from approximately 10- to 11-fold, the composite leverages the high atomic numbers of bismuth and tungsten to maintain efficient photon interaction even as the dominant mechanism transitions from photoelectric absorption to Compton scattering. Furthermore, integrating these oxides into a PEI polymer matrix provides additional advantages in terms of mechanical flexibility, reduced weight, ease of processing, and lower toxicity. These characteristics make the PEI-Bi_2_O_3_-WO_3_ system a promising candidate for next-generation radiation shielding applications in medical applications.

#### 3.2.3. Pair Production in the Nuclear Field

**Figure 5:**
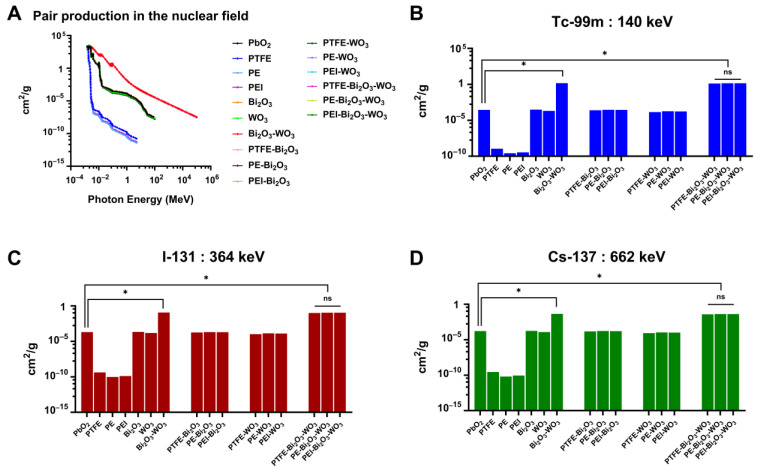
XCOM-based computational analysis of mass attenuation coefficients (cm^2^/g) for pair production in the nuclear field in bismuth oxide and tungsten oxide-reinforced polymer composites for gamma radiation shielding. (**A**) Comparison of pair production in the nuclear field cross-sections as a function of photon energy (MeV) for various materials including PbO_2_, PTFE, PE, PEI, Bi_2_O_3_, WO_3_, Bi_2_O_3_-WO_3_ composite, and reinforced polymer composites (PTFE-WO_3_, PE-WO_3_, PEI-WO_3_, PTFE-Bi_2_O_3_, PE-Bi_2_O_3_, PEI-Bi_2_O_3_, PTFE-Bi_2_O_3_-WO_3_, PE-Bi_2_O_3_-WO_3_, PEI-Bi_2_O_3_-WO_3_). (**B**–**D**) Bar graphs showing mass attenuation coefficients for pair production in the nuclear field at specific photon energies: (**B**) Tc-99m (140 keV), (**C**) I-131 (364 keV), and (**D**) Cs-137 (662 keV). Statistical significance is denoted as * *p* < 0.05; results are considered not significant when *p* > 0.05 (ns).

The MAC for pair production in the nuclear field, calculated via the XCOM database, are presented in Figure 5. This analysis evaluates the photon interaction characteristics of Bi_2_O_3_ and WO_3_ reinforced polymer composites, focusing on their applicability for gamma radiation shielding across a range of photon energies. As shown in Figure 5A, the cross-section curves of pair production for all studied materials increase progressively with rising photon energy, reflecting the energy dependence of this interaction process. Pair production becomes energetically feasible only above the threshold of 1.022 MeV, corresponding to twice the rest mass energy of an electron. However, the plotted range begins below this threshold to illustrate the trend across photon energies comprehensively. Materials containing high-Z elements, notably PbO_2_ and Bi_2_O_3_-based composites, exhibit distinctly higher pair production cross-sections, attributable to the proportionality of pair production to the square of the atomic number (Z^2^). In comparison, pure polymer matrices such as PTFE, PE, and PEI demonstrate significantly lower cross-sections, as expected due to their lower average atomic numbers and densities. Among the polymer composites, those containing both Bi_2_O_3_ and WO_3_, such as PEI-Bi_2_O_3_-WO_3_, consistently outperform the single-filler composites across the photon energy spectrum, indicating a synergistic effect from the combination of high-Z fillers.

Notable observations are evident when considering specific photon energies corresponding to widely used radionuclides. At 140 keV, corresponding to the gamma emission of Tc-99m (Figure 5B), among all tested materials, the PEI-Bi_2_O_3_-WO_3_ composite exhibits an exceptionally high attenuation coefficient of 1.41 cm^2^/g, whereas traditional PbO_2_ shows a markedly lower value of 2.80 × 10^−4^ cm^2^/g, indicating a ~5036-fold increase in interaction probability. These findings further affirm the PEI-Bi_2_O_3_-WO_3_ composite’s exceptional shielding performance, positioning it as a highly efficient and advanced material for radiation protection.

At 364 keV, characteristic of I-131 gamma emissions (Figure 5C), the PEI-Bi_2_O_3_-WO_3_ composite demonstrates a remarkably high attenuation coefficient of 0.109 cm^2^/g, vastly surpassing the conventional benchmark material PbO_2_, which records only 2.02 × 10^−4^ cm^2^/g—representing a substantial ~540-fold improvement in attenuation efficiency.

At 662 keV, corresponding to Cs-137 (Figure 5D), the PEI-Bi_2_O_3_-WO_3_ composite continues to demonstrate its potential. With a MAC of 4.15 × 10^−2^ cm^2^/g, significantly higher than PbO_2_ at 1.65 × 10^−4^ cm^2^/g, it offers a ~251-fold improvement. This enhanced performance, driven by the impact of high-Z elements (Bi and W), underscores the composite’s potential as a highly effective, lead-free shielding material for broad-spectrum gamma radiation protection.

As a result, the comparative analysis of MAC at photon energies corresponding to key medical radionuclides—Tc-99m, I-131, and Cs-137—demonstrates the superior radiation shielding performance of the PEI-Bi_2_O_3_-WO_3_ composite over conventional PbO_2_. The composite consistently exhibits significantly higher attenuation coefficients at each energy level, with fold increases of approximately 5036, 540, and 251, respectively. These substantial improvements are attributed to the high-Z elements (Bi and W), which enhance photon interaction probabilities across all energy ranges. Even at sub-threshold energies for pair production, the composite shows strong attenuation, emphasizing its robustness. Collectively, these findings validate the PEI-Bi_2_O_3_-WO_3_ composite as a highly effective, lead-free material for broad-spectrum gamma radiation shielding.

#### 3.2.4. Total Attenuation Without Coherent Scattering

**Figure 6:**
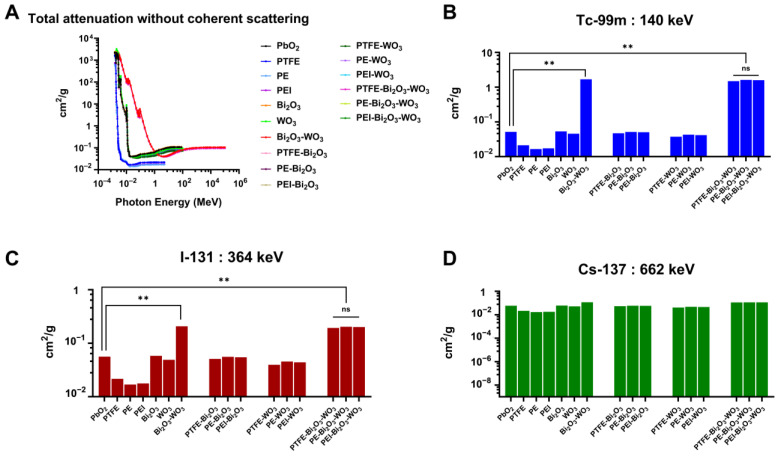
XCOM-based computational analysis of mass attenuation coefficients (cm^2^/g) for total attenuation without coherent scattering in bismuth oxide and tungsten oxide-reinforced polymer composites for gamma radiation shielding. (**A**) Comparison of total attenuation without coherent scattering cross-sections as a function of photon energy (MeV) for various materials including PbO_2_, PTFE, PE, PEI, Bi_2_O_3_, WO_3_, Bi_2_O_3_-WO_3_ composite, and reinforced polymer composites (PTFE-WO_3_, PE-WO_3_, PEI-WO_3_, PTFE-Bi_2_O_3_, PE-Bi_2_O_3_, PEI-Bi_2_O_3_, PTFE-Bi_2_O_3_-WO_3_, PE-Bi_2_O_3_-WO_3_, PEI-Bi_2_O_3_-WO_3_). (**B**–**D**) Bar graphs showing mass attenuation coefficients for total attenuation without coherent scattering at specific photon energies: (**B**) Tc-99m (140 keV), (**C**) I-131 (364 keV), and (**D**) Cs-137 (662 keV). Statistical significance is denoted as ** *p* < 0.01; results are considered not significant when *p* > 0.05 (ns).

Figure 6 illustrates the total attenuation without coherent scattering MAC for Bi_2_O_3_- and WO_3_-reinforced polymer composites. The attenuation decreases with increasing photon energy (Figure 6A), with PbO_2_ and Bi_2_O_3_-based composites consistently outperforming unfilled polymers. The inclusion of WO_3_, particularly in Bi_2_O_3_-WO_3_ hybrid systems, further enhances attenuation, with PEI-Bi_2_O_3_-WO_3_ achieving the highest performance.

At 140 keV (Figure 6B), composites incorporating Bi_2_O_3_ and WO_3_ fillers demonstrate a pronounced enhancement in attenuation compared to both unfilled polymers and conventional shielding materials such as PbO_2_. The PEI-Bi_2_O_3_-WO_3_ composite exhibited a MAC of 1.59 cm^2^/g, corresponding to an approximate 30.90-fold increase relative to PbO_2_ (5.15 × 10^−2^ cm^2^/g), with the improvement being statistically significant (*p* < 0.01). All Bi_2_O_3_-WO_3_ hybrid systems outperformed their single-filler and unfilled counterparts; however, no statistically significant differences (ns) were observed among the different polymer matrices containing the Bi_2_O_3_-WO_3_ hybrid. At 364 keV (Figure 6C), the overall attenuation efficiency decreases, consistent with the diminished contribution of the photoelectric effect at higher photon energies. Nevertheless, Bi_2_O_3_-WO_3_ composites continue to show enhanced MAC values compared to PbO_2_ and unfilled systems. The PEI-Bi_2_O_3_-WO_3_ composite achieved a MAC of 0.20 cm^2^/g, indicating a 3.58-fold improvement over PbO_2_ (5.61 × 10^−2^ cm^2^/g), with the difference remaining statistically significant (*p* < 0.05). The comparable performance of Bi_2_O_3_-WO_3_ composites across different polymer matrices reinforces the effectiveness of this hybrid filler approach in gamma attenuation. At the higher investigated photon energy of 662 keV (Figure 6D), emitted by Cs-137, attenuation efficiency further declines across all samples due to the dominance of Compton scattering. The PEI-Bi_2_O_3_-WO_3_ composite showed a MAC of 0.12 cm^2^/g, approximately 1.94 times greater than PbO_2_ (5.92 × 10^−2^ cm^2^/g). Although the differences among materials are less distinct at this energy level, Bi_2_O_3_-WO_3_-based composites consistently demonstrate improved attenuation compared to conventional formulations, even under high-energy conditions.

Consequently, these findings highlight the consistent advantage of incorporating Bi_2_O_3_-WO_3_ hybrid fillers into polymer matrices for enhanced gamma radiation shielding across a broad energy spectrum. The sustained attenuation performance across various host polymers demonstrates the versatility and adaptability of these composite systems. Furthermore, incorporating non-toxic, high-Z fillers offers a promising strategy for the development of lightweight, lead-free shielding materials that provide design flexibility. Enabling the optimization of mechanical and ergonomic properties without compromising radiation protection—an essential consideration for occupational safety in nuclear medicine and other radiation-intensive environments.

### 3.3. Advanced Computational Assessment of Gamma Photon Shielding Properties in Bi_2_O_3_- and WO_3_-Enhanced Polymer Composites Using Phy-X/PSD for Nuclear Medicine Safety

#### 3.3.1. Linear Attenuation Coefficients (LAC)

**Figure 7:**
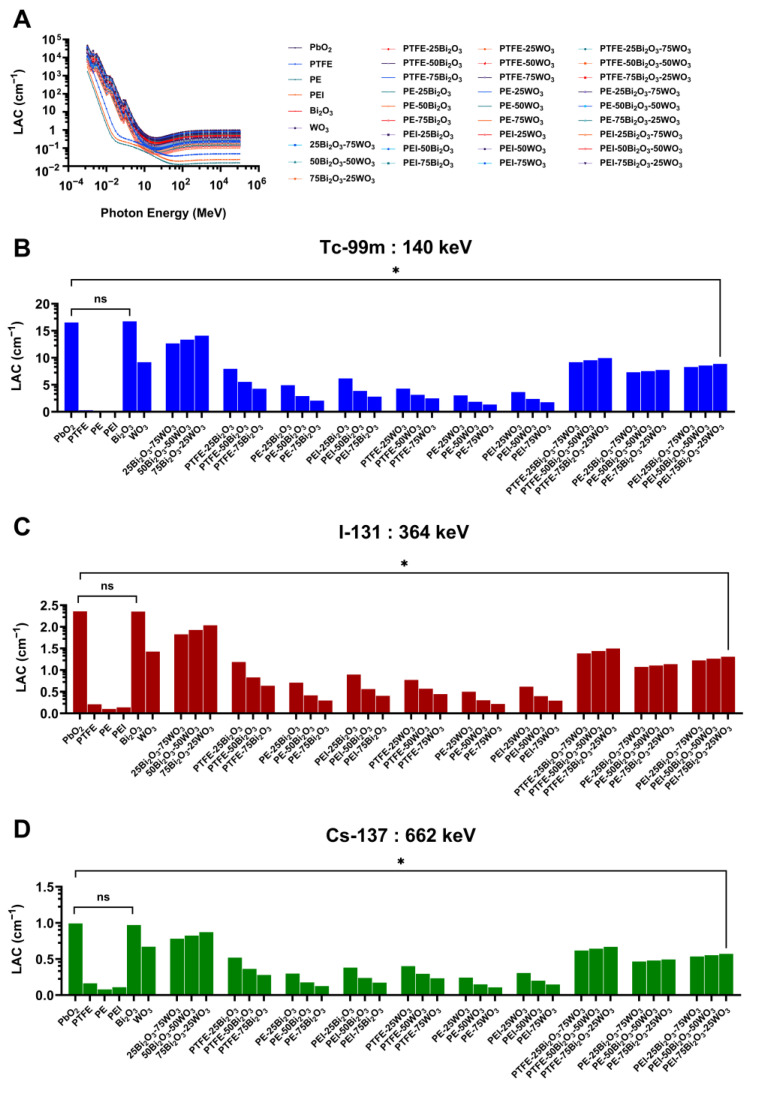
Computational analysis of linear attenuation coefficients (LAC, cm−1) in Bi_2_O_3_- and WO_3_-reinforced polymer composites for gamma radiation shielding in nuclear medicine. LAC values were calculated using the Phy-X/PSD software to assess the photon attenuation capabilities of various pure materials, binary oxides, and oxide-loaded polymer composites across a wide energy range and at energies relevant to nuclear medicine. (**A**) Energy-dependent LAC profiles of PbO_2_, Bi_2_O_3_, WO_3_, base polymers (PTFE, PE, PEI), and their oxide-reinforced composites are shown over a photon energy range from 1 keV to 10 MeV. (**B**–**D**) Bar charts depict the comparative LAC values at discrete gamma energies of (**B**) Tc-99m (140 keV), (**C**) I-131 (364 keV), and (**D**) Cs-137 (662 keV). Statistical comparisons indicate significant differences (*) between oxide-filled and pure polymer materials (*p* < 0.05), with “ns” denoting non-significant differences among base materials (*p* > 0.05).

The LAC, a fundamental parameter in radiation shielding studies, represents the material’s ability to attenuate photon beams as a function of both its atomic composition and density. In this study, LAC values for a wide array of Bi_2_O_3_- and WO_3_-reinforced polymer composites were calculated using the Phy-X/PSD software available at https://www.researchgate.net/publication/335993372_Phy-X_PSD_Development_of_a_user_friendly_online_software_for_calculation_of_parameters_relevant_to_radiation_shielding_and_dosimetry (accessed date 3 March 2025) and assessed over a broad photon energy range. The goal was to evaluate the shielding effectiveness of these composites, particularly at energy levels pertinent to nuclear medicine, including gamma emissions from Tc-99m, I-131, and Cs-137. Figure 7 summarizes the computational findings and highlights the impact of oxide reinforcement and polymer matrix selection on photon attenuation behavior.

Figure 7A presents the energy-dependent LAC values for pure oxides (PbO_2_, Bi_2_O_3_, WO_3_), base polymers (PTFE, PE, PEI), and various composite formulations. All materials exhibit significantly higher LAC values in the low-energy photon range (10^−2^ to 1 MeV), primarily due to the dominance of the photoelectric effect. With increasing photon energy, the LAC values sharply decline as the probability of photon-matter interactions diminishes, transitioning toward Compton scattering and pair production processes. Among the pure substances, PbO_2_ consistently demonstrates the highest LAC values, followed closely by Bi_2_O_3_. These results align with the higher atomic numbers and densities of these materials, which directly enhance photon attenuation. Conversely, the base polymers—PTFE, PE, and PEI—display low attenuation capacities, rendering them insufficient for gamma shielding in their native form. However, the addition of Bi_2_O_3_ and WO_3_ significantly elevates the LAC of polymer matrices, validating the efficacy of heavy metal oxide reinforcement in boosting radiation shielding properties.

At 140 keV (Figure 7B), corresponding to the gamma emission of Tc-99m widely used in diagnostic nuclear imaging, the shielding performance of the composites is more distinguishable. PbO_2_ displays the highest LAC (~16 cm^−1^), reflecting its superior photon absorption potential. Similarly, Bi_2_O_3_ and its oxide-polymer composites exhibit elevated LAC values, particularly when the oxide loading exceeds 50 wt%. The ternary composites, such as PE-50Bi_2_O_3_-50WO_3_ and PEI-75Bi_2_O_3_-25WO_3_, outperform both the base polymers and single-oxide reinforced systems. Statistical comparisons reveal significant differences (denoted by *) between oxide-enhanced composites and unfilled polymers, while no statistically significant (ns) difference exists among the base polymers themselves. These observations confirm that composite formulations containing both Bi_2_O_3_ and WO_3_ offer a highly effective and flexible approach to gamma radiation shielding at diagnostic energy levels.

At 364 keV (Figure 7C), representative of I-131 emissions used in both diagnostic and therapeutic nuclear medicine, the LAC values for all materials decline, consistent with the reduced interaction probability at higher photon energies. Nonetheless, Bi_2_O_3_-based composites continue to exhibit superior attenuation characteristics. Notably, PEI and PE matrices filled with high Bi_2_O_3_ content maintain LAC values around 1.5–2.0 cm^−1^, emphasizing their sustained shielding efficacy. Composites incorporating both Bi_2_O_3_ and WO_3_—such as PE-50Bi_2_O_3_-50WO_3_ and PEI-75Bi_2_O_3_-25WO_3_—achieve comparable or even improved LAC values, suggesting a synergistic interaction between the two oxides. This behavior demonstrates the capacity of these hybrid systems to attenuate intermediate-energy photons effectively, making them viable candidates for shielding in therapeutic nuclear procedures involving I-131.

At the highest photon energy examined—662 keV, associated with Cs-137 (Figure 7D)—a further reduction in LAC is observed across all materials due to the increasing dominance of Compton scattering. Despite this general decline, composites with high Bi_2_O_3_ loading remain among the top-performing materials. Specifically, PE-75Bi_2_O_3_-25WO_3_ and PEI-75Bi_2_O_3_-25WO_3_ maintain LAC values in the range of 0.8–1.0 cm^−1^, significantly outperforming base polymers, which remain below 0.2 cm^−1^. The consistent superiority of Bi_2_O_3_-enriched composites at high energy levels suggests that these materials can provide effective shielding not only for low- and mid-energy diagnostics but also for higher-energy radiological protection scenarios, such as external beam therapy or environmental radiation exposure.

An important observation across all energies is the relatively consistent enhancement in LAC provided by Bi_2_O_3_ in comparison to WO_3_. This can be attributed to Bi’s higher atomic number (Z = 83) and density, both of which substantially increase the probability of photon absorption via the photoelectric effect and scattering interactions. Furthermore, PEI-based composites frequently exhibit marginally higher LAC values compared to PTFE or PE-based counterparts, possibly due to differences in polymer density or oxide dispersion quality. The combination of Bi_2_O_3_ and WO_3_ within the same matrix also appears to leverage the individual advantages of both oxides, enhancing overall shielding while maintaining material flexibility and potentially improving structural properties.

As a result, this comprehensive computational analysis affirms that Bi_2_O_3_- and WO_3_-reinforced polymer composites offer substantial improvements in gamma radiation shielding performance across all energy levels relevant to nuclear medicine. The LAC demonstrate that these composites, particularly those with hybrid oxide reinforcement and high filler content, are highly effective in attenuating gamma photons.

#### 3.3.2. Half-Value Layer (HVL)

The HVL is a critical parameter in assessing the efficiency of radiation shielding materials, as it represents the thickness of a substance required to attenuate gamma radiation intensity by 50%. Materials with lower HVL values are more effective in shielding applications, as they require reduced thickness to provide the same level of protection. This is especially important in environments where material weight, space constraints, and flexibility are limiting factors. In this study, the HVL values of various Bi_2_O_3_- and WO_3_-reinforced polymer composites were evaluated using the Phy-X/PSD computational platform. The simulations covered a broad photon energy range (1 keV to 10 MeV), with particular attention to gamma energies relevant to nuclear medicine, namely Tc-99m, I-131, and Cs-137.

**Figure 8:**
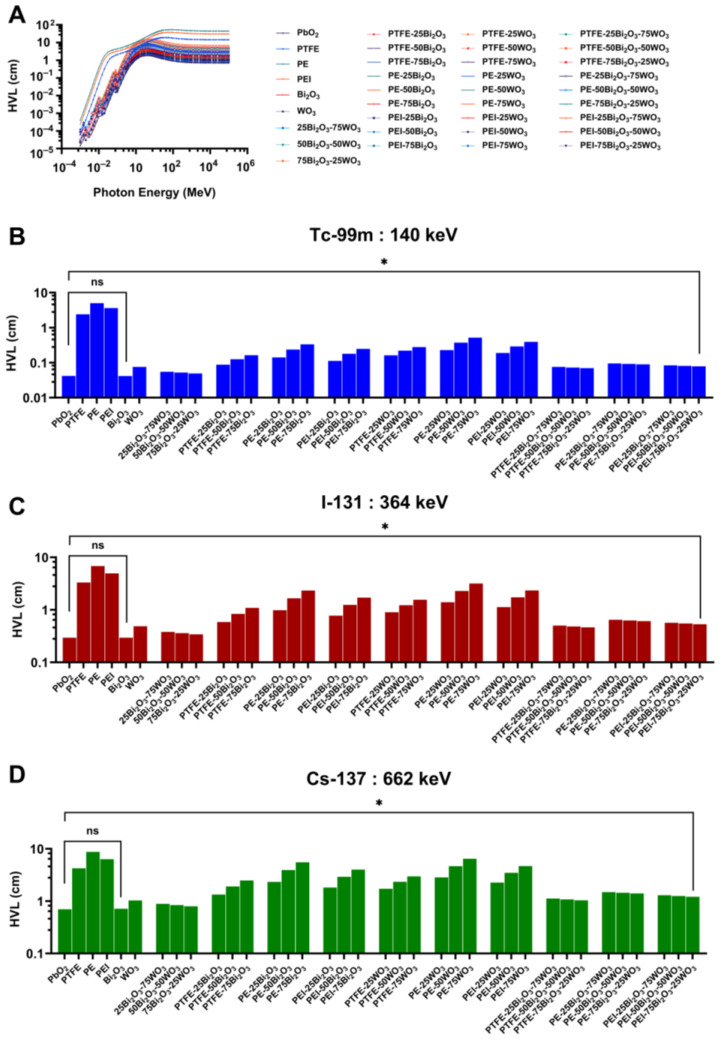
Computational evaluation of half-value layer (HVL, cm) for Bi_2_O_3_- and WO_3_-reinforced polymer composites as gamma radiation shields in nuclear medicine applications. The HVL, representing the material thickness required to reduce incident gamma radiation intensity by 50%, was calculated using Phy-X/PSD for various oxide-loaded polymer composites and benchmark materials. (**A**) Energy-dependent HVL values were assessed across a broad photon energy range (1 keV to 20 MeV) for pure compounds (PbO_2_, Bi_2_O_3_, WO_3_), base polymers (PTFE, PE, PEI), and their respective composites. (**B**–**D**) Bar graphs illustrate HVL values at specific photon energies relevant to nuclear medicine radionuclides: (**B**) Tc-99m (140 keV), (**C**) I-131 (364 keV), and (**D**) Cs-137 (662 keV). Statistical comparisons denote significant differences (*) between high-performance composites and base materials (*p* < 0.05), while “ns” indicates non-significant differences among certain unreinforced polymers (*p* > 0.05).

Figure 8A demonstrates that HVL values increase with photon energy across all materials, consistent with expected behavior. This trend indicates at lower energies a transition from photoelectric absorption dominance and at intermediate energies Compton scattering, and pair production at higher energies. Among the evaluated materials, high-Z oxides such as PbO_2_ and Bi_2_O_3_ exhibit significantly lower HVL values over the whole energy range, demonstrating their superior attenuation capability. In contrast, the unmodified polymer matrices—PTFE, PE, and PEI—show substantially higher HVL values, rendering them ineffective as standalone shielding materials. However, when these polymers are reinforced with high-Z oxides, particularly Bi_2_O_3_ and WO_3_, the HVL values decrease markedly, indicating a considerable enhancement in shielding performance.

Apparent differences in material performance emerge at 140 keV, corresponding to gamma emissions from Tc-99m commonly used in diagnostic nuclear medicine (Figure 8B). PbO_2_ displays the lowest HVL, followed closely by Bi_2_O_3_. Among the composites, those containing high proportions of Bi_2_O_3_ and/or WO_3_ show significantly reduced HVL values compared to the base polymers. Notably, composites such as PE-50Bi_2_O_3_-50WO_3_, PEI-50Bi_2_O_3_-50WO_3_, and PEI-75Bi_2_O_3_-25WO_3_ exhibit HVL values below 0.5 cm, demonstrating high attenuation efficiency at this diagnostic energy level. In contrast, unfilled polymers require material thickness exceeding several centimeters to achieve equivalent attenuation. These findings highlight the substantial benefits of high-Z filler inclusion for reducing shielding thickness without compromising performance.

At 364 keV, corresponding to I-131 emissions used in diagnostic and therapeutic nuclear medicine, the overall HVL values increase as expected due to the decreased likelihood of photon interaction at this energy level (Figure 8C). Nevertheless, the performance hierarchy remains consistent. Bi_2_O_3_-enriched composites, particularly those also containing WO_3_, maintain superior attenuation properties. For instance, PE-75Bi_2_O_3_-25WO_3_ and PEI-75Bi_2_O_3_-25WO_3_ composites achieve HVL values in the range of 0.5–1.0 cm, significantly outperforming unmodified polymer matrices, which display HVL values ranging from 5 to over 10 cm. These results confirm the sustained efficacy of high-Z oxide reinforcement even at intermediate photon energies, affirming their suitability for shielding applications in therapeutic procedures involving I-131.

At the highest investigated photon energy of 662 keV, corresponding to Cs-137 emissions (Figure 8D), the HVL values further increase across all material systems. Despite this trend, reinforced composites outperform base polymers by a significant margin. Composites such as PEI-75Bi_2_O_3_-25WO_3_ and PE-75Bi_2_O_3_-25WO_3_ maintain HVL values around 1.0–1.5 cm, while PTFE, PE, and PEI alone require 8–10 cm or more of material to achieve comparable attenuation. These differences are of critical importance in practical settings, as they directly impact the size, weight, and comfort of shielding products such as garments or portable barriers. The ability of oxide-filled composites to maintain high attenuation efficiency across a broad energy spectrum, including high-energy gamma photons, indicates their strong potential as advanced shielding materials for diagnostic and therapeutic applications.

A cross-comparison of all results highlights the clear advantage of Bi_2_O_3_ over WO_3_ in reducing HVL, attributable to Bi’s higher atomic number and density, which increase the probability of photon interaction, particularly via the photoelectric effect. Furthermore, incorporating both oxides within the same matrix often yields superior or comparable performance to single oxide systems, suggesting a synergistic effect that improves shielding and enhances mechanical stability, thermal resistance, and processability. PEI-based composites often performed slightly better than their PTFE and PE counterparts, likely due to higher intrinsic density or improved dispersion of oxide particles within the matrix.

#### 3.3.3. Tenth-Value Layer (TVL)

The TVL is a critical shielding parameter that quantifies the thickness of material required to attenuate gamma-ray intensity by 90%. Unlike the HVL, which reduces radiation to 50%, the TVL offers a more conservative and stringent benchmark for evaluating material efficiency, especially in high-dose or occupational settings where safety margins must be maximized. This study used a comprehensive computational analysis using Phy-X/PSD to determine the TVL of various polymer composites reinforced with Bi_2_O_3_ and WO_3_. The evaluation spanned a wide photon energy range (1 keV to 10 MeV) and focused particularly on discrete gamma energies of clinical relevance: Tc-99m (140 keV), I-131 (364 keV), and Cs-137 (662 keV). The resulting trends provide crucial insights into the attenuation capabilities and material optimization potential for radiation shielding applications in nuclear medicine.

**Figure 9:**
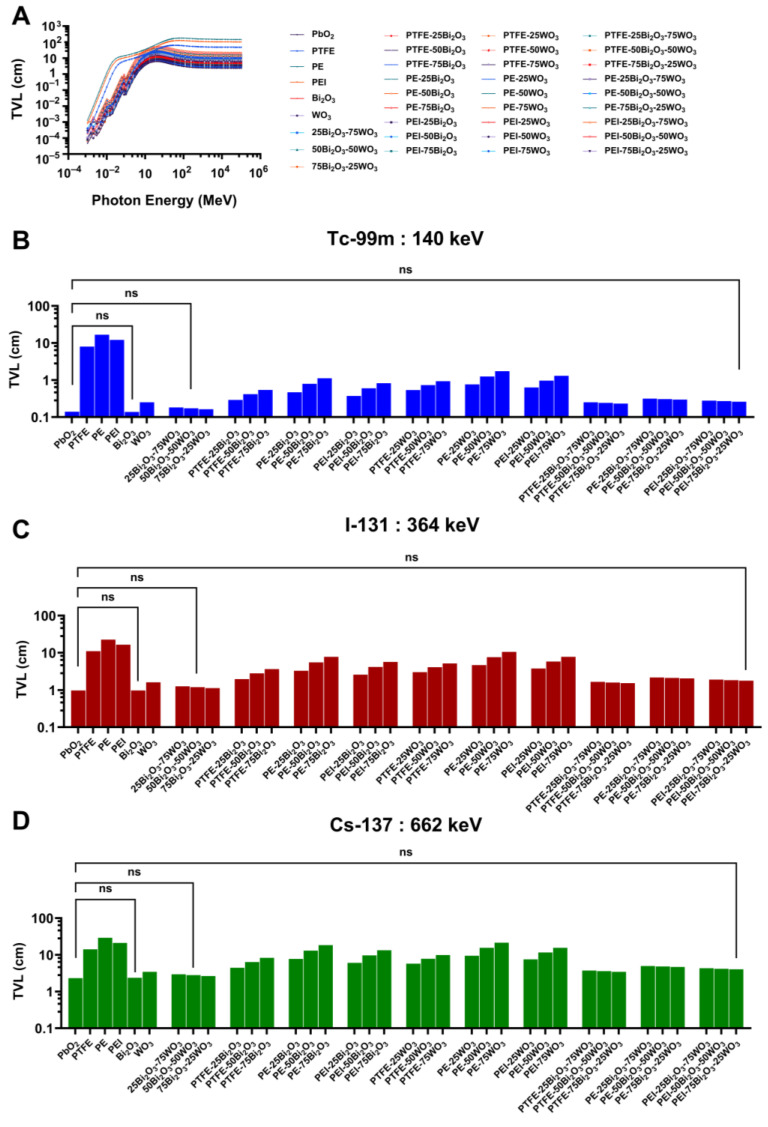
Computational assessment of tenth-value layer (TVL, cm) for Bi_2_O_3_- and WO_3_-reinforced polymer composites for gamma radiation shielding in nuclear medicine applications. The TVL, defined as the material thickness required to attenuate the incident gamma-ray intensity by 90%, was computed using the Phy-X/PSD platform for various pure substances, binary oxides, and polymer-based composites across a wide photon energy spectrum. (**A**) TVL as a function of photon energy (1 keV to 10 MeV) is shown for unmodified polymers (PTFE, PE, PEI), high-Z oxides (PbO_2_, Bi_2_O_3_, WO_3_), and a comprehensive series of Bi_2_O_3_/WO_3_-reinforced polymer composites. (**B**–**D**) Bar graphs represent TVL values at key photon energies used in nuclear medicine: (**B**) Tc-99m (140 keV), (**C**) I-131 (364 keV), and (**D**) Cs-137 (662 keV). Statistical comparisons show non-significant differences (ns) among base polymers (*p* > 0.05).

Figure 9A illustrates the energy-dependent behavior of TVL for all materials tested. In this study, the TVL increases with rising photon energy across the board. This behavior corresponds to the underlying physics of photon-matter interactions. At lower photon energies, the photoelectric effect dominates, resulting in higher probabilities of photon absorption and, consequently, lower required thicknesses to achieve 90% attenuation. However, as photon energy increases, the interaction cross-section diminishes due to the transition to Compton scattering and pair production at even higher energies, leading to a pronounced increase in TVL. Among the tested substances, PbO_2_ and Bi_2_O_3_ consistently exhibit the lowest TVL values, attributable to their high atomic numbers (Z = 82 for Pb and Z = 83 for Bi) and densities, enhancing their photon interaction efficiency. In contrast, the base polymer matrices—PTFE, PE, and PEI—demonstrate extremely high TVL values, exceeding >50 cm, thereby confirming their inadequacy as standalone shielding materials for gamma photons.

At 140 keV, corresponding to gamma emissions from Tc-99m, the performance disparity among materials becomes particularly stark (Figure 9B). PbO_2_ and Bi_2_O, yield minimal TVL values, well below 0.5 cm, due to their strong photoelectric absorption capabilities at this energy level. By comparison, unmodified polymers such as PTFE and PE display TVL values exceeding 10–50 cm, rendering them impractical for medical shielding without significant modification. However, when these polymers are reinforced with high-Z fillers, particularly Bi_2_O_3_ and WO_3_ in proportions of 25–75 wt%, the TVL drops dramatically. Composites such as PE-50Bi_2_O_3_-50WO_3_ and PEI-75Bi_2_O_3_-25WO_3_ achieve TVL values under 0.30 cm—comparable to those of pure metal oxides—highlighting their suitability as lightweight alternatives to traditional lead-based shielding. Statistical comparisons indicate no significant differences (ns) among the base polymers, yet a clear and meaningful improvement is observed with the introduction of oxide reinforcement.

At an intermediate photon energy of 364 keV, relevant to I-131, the overall attenuation efficiency decreases, leading to increased TVL values across all materials (Figure 9C). Nonetheless, the composite systems that performed well at lower energies continue to offer effective attenuation. High-performance materials such as PEI-75Bi_2_O_3_-25WO_3_ and PE-75Bi_2_O_3_-25WO_3_ maintain TVL values in the 2–4 cm range. These values are significantly lower than those of unmodified polymers, which require shielding thicknesses approaching or exceeding 30 cm. The findings highlight the significance of dual oxide incorporation in preserving shielding efficacy at energy levels where Compton scattering is the dominant interaction mechanism. Furthermore, achieving effective attenuation with reduced material thickness enhances the ergonomics and manufacturability of protective equipment, which is particularly advantageous for wearable applications, such as lead-free aprons and portable shielding panels.

At 662 keV, the energy associated with Cs-137, the shielding challenge becomes even more pronounced (Figure 9D). The need for dense, high-performance materials is most acute at this high energy, where photon interaction probabilities are lowest. Base polymers again fail to meet practical thresholds, requiring thicknesses well beyond 30–50 cm to achieve 90% attenuation. However, composites with high loadings of Bi_2_O_3_ and WO_3_—notably PE-50Bi_2_O_3_-50WO_3_ and PEI-75Bi_2_O_3_-25WO_3_—maintain relatively low TVL values, generally below 5 cm. This reduction in required thickness directly translates to substantial weight savings and increased design flexibility, making these composites highly favorable for protective systems where weight, mobility, and comfort are essential factors.Consequently, an important consideration across all three photon energies is the c11comparative advantage of Bi_2_O_3_ over WO_3_ as a reinforcing filler. Due to its higher atomic number and density, Bi_2_O_3_ consistently results in lower TVL values across all energy levels. However, the combination of Bi_2_O_3_ and WO_3_ often yields a synergistic effect, enhancing not only attenuation but also the mechanical integrity and thermal stability of the composite. Furthermore, among the three polymer matrices evaluated, PEI-based composites generally perform better than their PTFE and PE counterparts. This may be attributed to the higher density of the PEI matrix and its potential for superior filler dispersion, resulting in more homogeneous composite structures that support effective photon interaction and absorption.

#### 3.3.4. Mean Free Path (MFP)

The MFP is a key parameter in the evaluation of radiation shielding materials, representing the average distance a photon travels within a substance before undergoing an interaction such as photoelectric absorption or Compton scattering. Materials with shorter MFPs are more effective attenuators, as photons are more likely to interact over shorter distances. In this study, the MFP values of a broad range of Bi_2_O_3_- and WO_3_-reinforced polymer composites were calculated using the Phy-X/PSD simulation. The analysis was conducted over an extensive energy range, with emphasis placed on photon energies pertinent to nuclear medicine applications.

**Figure 10:**
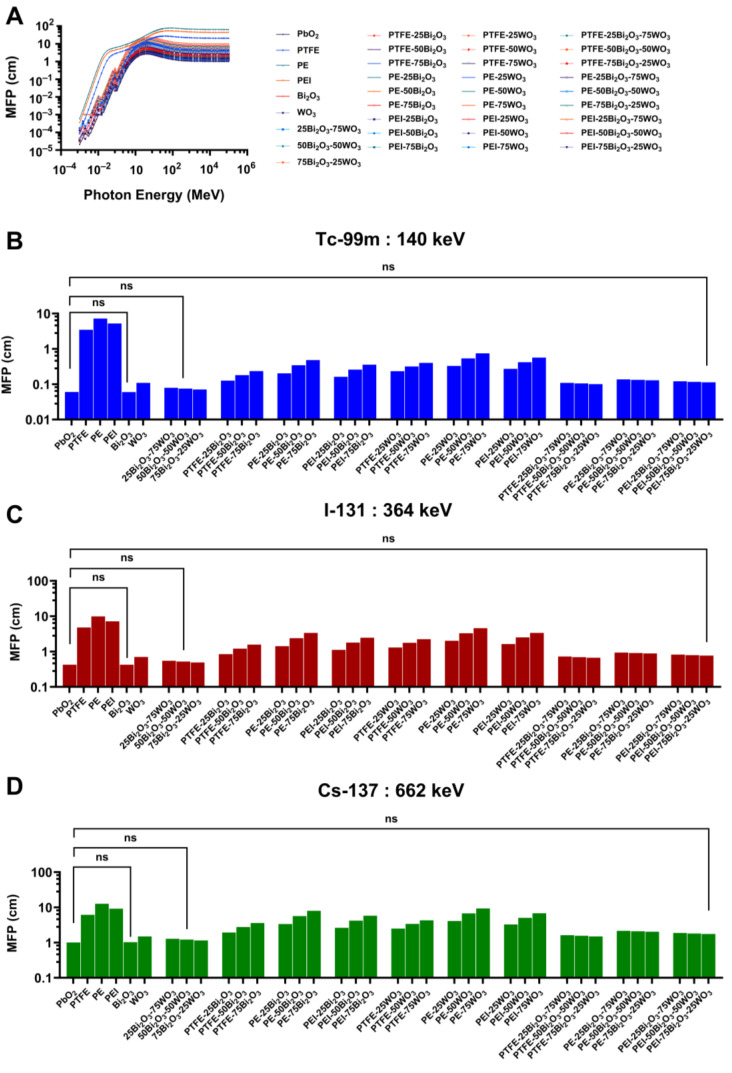
Computational evaluation of mean free path (MFP, cm) in Bi_2_O_3_- and WO_3_-reinforced polymer composites for gamma radiation shielding in nuclear medicine. (**A**) Energy-dependent MFP profiles are shown for base polymers (PTFE, PE, PEI), pure high-Z oxides (PbO_2_, Bi_2_O_3_, WO_3_), and a comprehensive series of polymer composites reinforced with Bi_2_O_3_ and/or WO_3_. (**B**–**D**) Bar plots illustrate MFP values at clinically relevant gamma energies: (**B**) Tc-99m (140 keV), (**C**) I-131 (364 keV), and (**D**) Cs-137 (662 keV). Statistical comparisons reveal non-significant differences (ns) among base polymers, whereas oxide-reinforced composites show marked in MFP (*p* > 0.05).

As illustrated in Figure 10A, MFP values for all materials increase as photon energy rises, consistent with the underlying physics of photon-matter interactions. At low photon energies, where the photoelectric effect predominates, interaction probabilities are high, resulting in short MFPs. As photon energy increases, the relative contribution of Compton scattering becomes dominant, and the interaction cross-section diminishes, causing MFP values to increase accordingly. High-Z materials such as PbO_2_ and Bi_2_O_3_ consistently exhibited the shortest MFPs across the energy spectrum, attributed to their high atomic numbers and densities, which enhance photon attenuation efficiency. In contrast, unmodified polymer matrices (PTFE, PE, and PEI) showed substantially longer MFPs, reflecting their limited capacity for gamma attenuation.

At 140 keV, which corresponds to the gamma emissions of Tc-99m, the contrast between composite and unfilled polymer materials is pronounced (Figure 10B). The base polymers exhibited MFP values exceeding 10 cm, indicative of inadequate shielding performance at this energy level. However, composites reinforced with high-Z oxides, particularly Bi_2_O_3_ and WO_3_ at ≥50 wt%, significantly improved photon interaction efficiency, reducing MFPs to below 0.5 cm in some formulations. Notably, PEI-75Bi_2_O_3_-25WO_3_ and PE-75Bi_2_O_3_-25WO_3_ achieved MFP values approaching those of pure Bi_2_O_3_ and PbO_2_. These results clearly demonstrate the impact of oxide reinforcement on enhancing photon attenuation properties. Statistical comparisons indicated no significant differences among the base polymers (ns), while the reduction in MFP for reinforced composites was substantial and of practical relevance.

At 364 keV, associated with I-131, MFP values increased across all materials, consistent with the shift from the photoelectric absorption to the Compton scattering as dominant interaction mechanism (Figure 10C). Despite this increase, oxide-reinforced composites continued to demonstrate marked improvements in attenuation performance compared to unmodified polymers. Composites such as PEI-75Bi_2_O_3_-25WO_3_ and PE-50Bi_2_O_3_-50WO_3_ exhibited MFPs in the range of 1 to 2 cm, which is significantly lower than the >10 cm MFPs observed in unreinforced matrices. This highlights the continued efficacy of high-Z filler incorporation at intermediate photon energies.

At 662 keV, corresponding to Cs-137 emissions, photon interaction probabilities are further reduced, resulting in generally higher MFP values across all tested materials (Figure 10D). However, the overall performance trend remains consistent. While unfilled polymers exhibit MFPs well above 50 cm, composites such as PEI-75Bi_2_O_3_-25WO_3_ and PE-75Bi_2_O_3_-25WO_3_ maintain significantly lower MFPs (approximately 5–10 cm), representing a substantial attenuation improvement. These results suggest that oxide-filled composites remain viable candidates for shielding even in high-energy photon environments.

Across all energy levels, Bi_2_O_3_ consistently outperformed WO_3_ in reducing MFP values, which is attributable to bismuth’s higher atomic number and mass attenuation coefficient. However, composite formulations incorporating both oxides demonstrated synergistic effects, combining the attenuation benefits of Bi_2_O_3_ with the structural and thermal stability enhancements afforded by WO_3_. Among the polymers assessed, PEI-based composites frequently exhibited the lowest MFP values, which may be attributed to higher matrix density, better compatibility with filler particles, or improved dispersion of the oxides within the matrix. These advantages further support PEI as a favorable base material for the development of advanced radiation shielding systems.

## 4. Discussion

The composite material properties are a key factor in determining the potential of composites for radiation shielding. Our simulated heat transfer profiles reveal that PTFE and PEI exhibit minimal thermal penetration (~2 mm) under high-temperature conditions, indicating superior thermal resistance compared to PE, which shows deeper heat diffusion (>6 mm) at lower melting temperatures (Figure 1). These findings, in line with Sayyed [38] and Wu et al. [39], suggest that high-performance polymers like PEI can maintain dimensional stability and suppress filler agglomeration under prolonged thermal loads, thereby enhancing the uniformity and durability of gamma shielding composites. Similarly, Yuan et al. [40] have shown that PTFE-based composites with Bi_2_O_3_ fillers exhibit stable shielding efficiency after repeated thermal cycling, confirming the material’s potential for reusable radiation protection garments. These findings highlight the importance of considering the thermal properties of composite materials in radiation shielding applications. On the other hand, while PE offers easy processing and moldability—beneficial for mass production of disposable shields—its lower thermal threshold may compromise long-term performance and filler integrity in high-radiation or sterilization-prone clinical settings, as observed by Chen et al. in low-density polyethylene (LDPE) blends [41].

The finite element results demonstrate that PEI exhibits significantly superior stiffness and dimensional stability under compressive loading compared to PTFE and PE, with maximum deformation remaining below 1 mm even at 100 N (Figure 2). These findings align with prior studies indicating the mechanical superiority of polyetherimide for load-bearing applications, particularly in radiation shielding systems where geometric integrity is critical for attenuation performance [42,43,44]. In contrast, the nonlinear deformation behavior of PTFE under load suggests its limitations in structural roles without reinforcement, despite its favorable chemical resistance [28,45,46].

The XCOM simulation results (Figure 3) demonstrate that the PEI-Bi_2_O_3_-WO_3_ composite exhibits outstanding gamma radiation shielding across all tested energies compared to conventional PbO_2_. At 140 keV (Tc-99m), the composite achieved a ~7000-fold higher MAC, while at 364 keV (I-131) and 662 keV (Cs-137), improvements of ~2063- and ~1370-fold were observed, respectively. Although MAC values decreased with increasing energy, the composite consistently outperformed PbO_2_, indicating robust energy-independent shielding. These findings align with previous studies [22,47,48], which also reported enhanced attenuation in high-Z-filled composites, particularly those incorporating Bi_2_O_3_, due to its high electron density and effective atomic number. The similar trends observed between incoherent (Compton) scattering and photoelectric absorption arise from their common dependence on photon energy, atomic number (Z), and electron density of the composite. High-Z fillers such as Bi_2_O_3_ and WO_3_ enhance both interactions. As photon energy increases, the contributions from photoelectric effect and Compton scattering decline, resulting in parallel attenuation patterns. This behavior reflects fundamental photon–matter interaction mechanisms and is particularly evident in engineered radiation shielding materials. The superior performance is attributed to the high-Z nature of Bi and W oxides enhancing scattering and absorption. The advantage of dual-filler systems (Bi_2_O_3_-WO_3_) was primarily evident at lower photon energies, diminishing as energy increased, emphasizing the need for energy-specific filler selection to optimize shielding efficacy. This novel finding, as documented in [49,50,51,52], and is a significant contribution to the field of materials science and radiation physics. Additionally, incorporating these oxides into a PEI matrix offers advantages in flexibility, reduced toxicity, and lower weight, making the composite highly suitable for modern, safer radiation shielding applications.

The superior photoelectric absorption and mass attenuation performance of Bi_2_O_3_-WO_3_ hybrid composites (Figure 4) particularly at clinically relevant photon energies such as 140 keV and 364 keV support the findings from prior studies demonstrating enhanced gamma shielding through high-Z filler synergy in polymer matrices. Consistent with reports by Alsaab and Zeghib [53] and Toyen et al. [54]. The PEI-Bi_2_O_3_-WO_3_ achieved 10- to 11-fold enhanced photoelectric absorption cross-sections than PbO_2_. These results confirm that filler composition plays a more decisive role than polymer matrix type in governing shielding efficacy at intermediate and high photon energies.

Pair production in the nuclear field analysis (Figure 5) demonstrated that while pair production plays a negligible role at photon energies relevant to common medical isotopes (e.g., Tc-99m, I-131, Cs-137), the inclusion of high-Z fillers such as Bi_2_O_3_ and WO_3_ significantly enhances the MAC, a measure of how a material attenuates the intensity of a beam of radiation, of polymer composites across the studied energy range. Consistent with previous findings [55,56,57]. Bi_2_O_3_-WO_3_ hybrid systems exhibited superior performance due to the synergistic effects of dual high-Z reinforcements, with minimal influence from the choice of polymer matrix. In comparison to PbO_2_ the PEI-Bi_2_O_3_-WO_3_ composite pair production attenuation resulted in fold increases of approximately 5036 at 140 keV, 540 at 364 keV, and 251 at 662 keV. These results align with established knowledge that pair production becomes significant only above 1.022 MeV. However, even sub-threshold photon energies benefit from high-Z additives in shielding materials [58]. These findings align with previous studies demonstrating the superior gamma shielding efficiency of Bi_2_O_3_- and WO_3_-loaded materials due to their high atomic numbers and synergistic interactions [59,60]. The consistent attenuation observed in Bi_2_O_3_-WO_3_ hybrids emphasizes their promise as effective, lightweight shielding alternatives for nuclear medicine applications and these findings align with previous reports demonstrating the superior shielding efficiency of high-Z fillers such as Bi_2_O_3_ and WO_3_ in polymer composites [61,62,63,64,65]. Notably, the non-significant influence of the polymer matrix reinforces that filler composition is the primary determinant of attenuation performance in such systems.

Moreover, at 140 keV, Bi_2_O_3_ and WO_3_ fillers show a significant enhancement in attenuation relative to PbO_2_, resulting in a theoretical 30.90-fold increase, a 3.58-fold improvement at 364 keV, and a 1.94-fold enhancement at 662 keV. The results indicate that Bi_2_O_3_- and Bi_2_O_3_-WO_3_-reinforced polymer composites exhibit significantly enhanced photon attenuation across a broad energy range, particularly at diagnostic and therapeutic photon energies (140–662 keV), outperforming unfilled polymers and WO_3_-only systems. These findings align with previous studies demonstrating the superior shielding performance of high-Z fillers like Bi_2_O_3_ in polymer matrices due to their high photoelectric absorption cross-sections [66,67]. Notably, the minimal influence of polymer type on attenuation is consistent with earlier reports, suggesting that filler composition is the primary determinant of shielding efficacy [68].

The Phy-X/PSD simulations (Section 3.3.) demonstrate that adding high-Z metal oxides into polymer matrices significantly enhances gamma-ray attenuation properties in the diagnostic and therapeutic energy range (140–662 keV), which is significant for nuclear medicine applications. Furthermore, Bi_2_O_3_ was the most efficient additive owing to its high atomic number (Z = 83) and density, which lowered HVL, TVL, and MFP values in comparison to WO_3_. The composites PE-50Bi_2_O_3_-50WO_3_ and PEI-75Bi_2_O_3_-25WO_3_ exhibited LAC, and shielding performance values that closely match those of pure metal oxides, particularly at 140 keV, which is relevant to Tc-99m, and displayed improved performance at higher energies typical of Cs-137 emissions. These findings align with earlier experimental and computational studies. El-Khayatt showed that bismuth-based glass systems had better gamma attenuation than tungsten analogs owing to their greater photoelectric cross-sections at diagnostic energy levels, which supports the present results [69]. Moreover, hybrid compositions combining Bi_2_O_3_ and WO_3_ provide synergistic benefits by guaranteeing efficient attenuation performance and improving mechanical and thermal qualities. The results show that dual-oxide systems efficiently balance shielding and structural performance, hence supporting those of Alajerami et al. [70]. Two main material properties, higher intrinsic matrix density and better oxide particle dispersion, both of which directly affect gamma-ray interaction probabilities inside the composite structure, explain the better attenuation performance seen in PEI-based composites compared to those derived from PTFE and PE. Moreover, our work shows that for uses in nuclear medicine and radiation protection, polymer composites packed with Bi_2_O_3_ and WO_3_ are efficient, lead-free, and lightweight shielding materials. Moreover, the results show that polymer composites loaded with Bi_2_O_3_ and WO_3_ are efficient, lead-free, lightweight shielding materials appropriate for radiation protection in nuclear medicine facilities.

As the incidence of ionizing radiation from medical applications increases, the potential for health risks also increases and cannot be disregarded. Furthermore, due to the uncertainties surrounding the risks associated with low radiation doses, a recent article in The Lancet highlighted the necessity of following the core principles of radiological protection and justifying diagnostic procedures that utilize ionizing radiation, asserting imaging should be conducted at the lowest possible dosage [71].

## 5. Conclusions

This study highlights the clinical relevance of Bi_2_O_3_- and WO_3_-reinforced polymer composites as efficient, lightweight, and lead-free materials for radiation shielding in medical environments. Simulations confirm that high-performance polymers like PEI provide superior thermal and mechanical stability, critical for maintaining structural integrity in reusable shielding garments and equipment. Bi_2_O_3_-based fillers, especially in hybrid systems with WO_3_, demonstrate excellent gamma-ray attenuation at diagnostic and therapeutic energy levels (140–662 keV), outperforming unfilled and WO_3_-only composites. These materials maintain low HVL and TVL values, ensuring effective protection against common medical isotopes such as Tc-99m and Cs-137.

Given the increasing clinical use of ionizing radiation and the associated safety concerns, these findings support the adoption of advanced polymer composites as practical, sustainable alternatives to traditional lead-based shielding, promoting safer and more ergonomic protection for both patients and healthcare professionals. Importantly, the ongoing advancements in nanotechnology and material science are continually enhancing the capabilities of polymer-based radiation shields, making them promising candidates for future use in healthcare. This potential opens up exciting possibilities for future applications in healthcare.

## Data Availability

The original contributions presented in this study are included in the article material. Further inquiries can be directed to the corresponding author.
